# Proteomic characterization of a lutein-hyperaccumulating *Chlamydomonas reinhardtii* mutant reveals photoprotection-related factors as targets for increasing cellular carotenoid content

**DOI:** 10.1186/s13068-023-02421-0

**Published:** 2023-11-04

**Authors:** Josie L. McQuillan, Edoardo Andrea Cutolo, Caroline Evans, Jagroop Pandhal

**Affiliations:** 1 Department of Chemical and Biological Engineering, University of Sheffield, Mappin Street, Sheffield, S1 3JD UK; 2 Laboratory of Photosynthesis and Bioenergy, Department of Biotechnology, University of Verona, Strada le Grazie 15, 37134 Verona, Italy

**Keywords:** Lutein, Norflurazon, Quantitative proteomics, *Chlamydomonas reinhardtii*, Photoprotection, Pigments, Light-harvesting complex stress-related proteins, Non-photochemical quenching, Microalgae

## Abstract

**Background:**

Microalgae are emerging hosts for the sustainable production of lutein, a high-value carotenoid; however, to be commercially competitive with existing systems, their capacity for lutein sequestration must be augmented. Previous attempts to boost microalgal lutein production have focussed on upregulating carotenoid biosynthetic enzymes, in part due to a lack of metabolic engineering targets for expanding lutein storage.

**Results:**

Here, we isolated a lutein hyper-producing mutant of the model green microalga *Chlamydomonas reinhardtii* and characterized the metabolic mechanisms driving its enhanced lutein accumulation using label-free quantitative proteomics. Norflurazon- and high light-resistant *C. reinhardtii* mutants were screened to yield four mutant lines that produced significantly more lutein per cell compared to the CC-125 parental strain. Mutant 5 (Mut-5) exhibited a 5.4-fold increase in lutein content per cell, which to our knowledge is the highest fold increase of lutein in *C. reinhardtii* resulting from mutagenesis or metabolic engineering so far. Comparative proteomics of Mut-5 against its parental strain CC-125 revealed an increased abundance of light-harvesting complex-like proteins involved in photoprotection, among differences in pigment biosynthesis, central carbon metabolism, and translation. Further characterization of Mut-5 under varying light conditions revealed constitutive overexpression of the photoprotective proteins light-harvesting complex stress-related 1 (LHCSR1) and LHCSR3 and PSII subunit S regardless of light intensity, and increased accrual of total chlorophyll and carotenoids as light intensity increased. Although the photosynthetic efficiency of Mut-5 was comparatively lower than CC-125, the amplitude of non-photochemical quenching responses of Mut-5 was 4.5-fold higher than in CC-125 at low irradiance.

**Conclusions:**

We used *C. reinhardtii* as a model green alga and identified light-harvesting complex-like proteins (among others) as potential metabolic engineering targets to enhance lutein accumulation in microalgae. These have the added value of imparting resistance to high light, although partially compromising photosynthetic efficiency. Further genetic characterization and engineering of Mut-5 could lead to the discovery of unknown players in photoprotective mechanisms and the development of a potent microalgal lutein production system.

**Supplementary Information:**

The online version contains supplementary material available at 10.1186/s13068-023-02421-0.

## Background

Eukaryotic microalgae are photosynthetic microorganisms capable of capturing and transforming light energy and carbon dioxide (CO_2_) into high-value products such as lipids, pigments, and proteins, among other useful compounds [1–3]. Of these, the yellow-orange carotenoid lutein is of particularly high value; it is a powerful antioxidant and an essential human dietary nutrient [4], providing pro-vitamin A and protecting against certain cancers, cardiovascular diseases, and age-related macular degeneration [5, 6]. Several microalgal species naturally accumulate high lutein contents, including two recently described trebouxiophyceae, *Parachlorella* sp. JD-076 and *Chlorella sorokiniana* FZU60, which produce up to 11.87 and 11.22 mg lutein/g dry cell weight (DCW), respectively [7, 8], and the chlorodendrophycea *Tetraselmis striata* CTP4 strain (3.81 mg g^−1^ DCW) [9]. Lutein is currently produced commercially by extracting oleoresin from the petals of *Tagetes erecta* (marigold plants), although microalgae could offer several advantages over marigold farming, including faster growth rates, reduced land, water and labour requirements, and less susceptibility to seasonal perturbations [10, 11]. Moreover, microalgal lutein is synthesized in free form, whereas marigold-derived lutein is esterified and requires an extra saponification processing step [11]. Although high levels of lutein production have recently been achieved by optimizing the growth parameters and extraction methods of some microalgal strains including those mentioned above [7, 8, 12, 13], applying a combination of strain selection, growth optimization, and metabolic engineering strategies could further increase productivity.

In microalgae, lutein is synthesized in plastids, where it is predominantly bound to light-harvesting complex (LHC) proteins, among other xanthophyll and chlorophyll molecules [14, 15]. Xanthophylls such as lutein participate in light-harvesting, are required for the proper assembly and structural organization of photosystem II (PSII), and confer high light tolerance by acting as quenchers of triplet-state chlorophyll, which is responsible for the production of damaging singlet oxygen radicals [16–18]. Due to its hydrophobic nature, lutein cannot accumulate freely in the chloroplast stroma and must be sequestered within membranes or enclosed hydrophobic environments; in microalgae, this is mostly limited to LHCs within the thylakoid membrane [14]. This presents a threshold to the amount of lutein that can accumulate, set by the number of LHCs present within the thylakoid [19]. Discovering a means to overcome this natural storage capacity barrier could therefore improve the commercial viability of lutein production in microalgae.

The green microalga *Chlamydomonas reinhardtii* naturally produces lutein; although not credited as a high lutein producer compared to other species [20], this model alga has the benefit of having fast growth, genetic tractability, and several decades’ worth of research and omics data [21]. Furthermore, the nuclear *C. reinhardtii* genome is haploid, meaning that all mutations are dominant, and *C. reinhardtii* can reproduce both asexually and sexually, enabling genetic crosses between strains exhibiting desirable characteristics, *i.e*. selective breeding. Improvements in lutein accumulation in *C. reinhardtii* may be translatable to other more productive microalgal species, although recent advances in *C. reinhardtii* scale-up suggest that this species may soon be a feasible industrial producer [22, 23].

Several attempts have been made to enhance carotenoid production in *C. reinhardtii*; in most cases, carotenoid biosynthesis has been targeted via overexpression of rate-limiting enzymes in the carotenogenesis pathway, such as the heterologous expression of phytoene desaturase from *Dunaliella salina* and *Chlorella zofingiensis*, leading to 2.6-fold and 2.2-fold increases in lutein content, respectively [24, 25]. Otherwise, various forms of the putative carotenoid biosynthesis regulator ORANGE were overexpressed in *C. reinhardtii*, generating 1.7–3.1-fold increases in lutein content [26–28]. Despite these increases, the amount of lutein produced by targeting its biosynthesis alone may, as mentioned above, be limited by the carotenoid storage capacity of the cells. Overexpressing individual enzymes in *C. reinhardtii* may also be hampered by notoriously stringent metabolic pathway regulation at multiple levels, including feedback inhibition [29–32]. A powerful strategy to rapidly generate new traits in microalgae, including pigment hyper-producing phenotypes, is random mutagenesis followed by stringent selection [33]. This method, which has already been applied successfully to enhance the production of lutein and other carotenoids in microalgae [20, 34, 35], also provides opportunity to discover novel characteristics within metabolic pathways and their regulation, and possibly new targets for metabolic engineering. Here, we generated a pigment hyperaccumulating *C. reinhardtii* mutant (Mut-5) by random chemical mutagenesis, using a selection process during which cells were simultaneously subjected to the carotenoid inhibitor norflurazon and high light. Mutants were analysed for lutein accumulation by high-performance liquid chromatography (HPLC), which revealed a very high-lutein phenotype in Mut-5. To acquire insights into the proteins and pathways responsible for the increased pigment storage of Mut-5, we performed comparative label-free quantitative (LFQ) proteomics, which identified specific LHC proteins among others as potential genetic engineering targets for enhancing lutein production in microalgae. We then performed biophysical and biochemical analyses on the new strain, generating mechanistic insight into positive and negative metabolic consequences of the high-pigment and constitutively active-photoprotection phenotype.

## Results

### Generation and characterization of Mut-5, a hyper-pigmented *C. reinhardtii* mutant

To randomly generate *C. reinhardtii* carotenoid-overproducing mutants, CC-125 cells were chemically mutagenized with ethyl methanesulfonate (EMS) and spread on to tris-acetate-phosphate (TAP) agar plates supplemented with the carotenoid biosynthesis inhibitor norflurazon, and then grown under high light (1050 ± 150 μmol photons m^2^ s^−1^). The combined effects of high light and norflurazon enhance individual negative effects on *C. reinhardtii* growth [36], due to the vital role of carotenoids in protecting cells from strong irradiation, which damages cells via the generation of reactive oxygen species [17, 37]. Carotenoid biosynthesis inhibitors such as norflurazon have successfully been used to isolate carotenoid overproducing microalgal mutants, although typically lethal concentrations of inhibitor were applied, leading to the isolation of strains carrying mutations in the target carotenoid enzyme [20, 38, 39]. To avoid restricting mutations to phytoene desaturase, the enzymatic target of norflurazon, sub-lethal concentrations of norflurazon that confer some but not total inhibition of phytoene desaturase were applied in this study, with the goal of revealing novel mechanisms that increase carotenoid production.

Of the 648 colonies that survived the combined selection pressures of norflurazon and high light, nine mutants exhibited significantly higher relative carotenoid contents under ambient conditions compared to the parental strain CC-125 at the final stage of screening (Additional File 1). These mutants were scaled up to 25 mL shake-flask cultures and grown under standard mixotrophic conditions for 96 h for pigment analysis by spectrophotometry and HPLC. Three of the mutants (Mut-5, Mut-6, and Mut-7) exhibited significantly higher total chlorophyll (Chl) and total carotenoid contents compared to CC-125, the highest being Mut-5, which accumulated 3.0-fold more total Chl and 3.6-fold more total carotenoids per cell than the CC-125 parental strain (Table 1). The lutein content of Mut-5 was also significantly higher than CC-125, and to a greater extent than the other mutants, exhibiting 5.4-fold and 2.3-fold higher lutein contents per cell and per g of dry cell weight, respectively (Table 1).

**Table 1:** Pigment contents of *C. reinhardtii* CC-125 and nine high light- and norflurazon-resistant mutant strains

Strain	Chl a (pg cell^−1^)	Chl b (pg cell^−1^)	Total Chl (pg cell^−1^)	Total Car (pg cell^−1^)	Lutein Content	
					fg cell^−1^	mg g^−1^ DCW
CC-125	1.13 ± 0.26	0.57 ± 0.12	1.70 ± 0.38	0.25 ± 0.05	151 ± 24	1.77 ± 0.18
Mut-1	1.15 ± 0.20	0.69 ± 0.08	1.84 ± 0.28	0.31 ± 0.07	206 ± 25	1.89 ± 0.08
Mut-2	1.84 ± 0.24	1.02 ± 0.11^*^	2.86 ± 0.35	0.45 ± 0.04	335 ± 7^*^	2.43 ± 0.07
Mut-3	0.76 ± 0.24	0.41 ± 0.15	1.18 ± 0.37	0.20 ± 0.06	133 ± 61	1.90 ± 0.66
Mut-4	1.25 ± 0.24	0.75 ± 0.15	1.99 ± 0.39	0.39 ± 0.06	281 ± 26	2.60 ± 0.12
Mut-5	3.44 ± 0.70^****^	1.74 ± 0.25^****^	5.18 ± 0.94^****^	0.92 ± 0.18^****^	806 ± 91^****^	4.13 ± 0.4^****^
Mut-6	2.67 ± 0.19^**^	1.50 ± 0.09^***^	4.16 ± 0.28^***^	0.65 ± 0.06^**^	468 ± 57^***^	2.97 ± 0.48^*^
Mut-7	2.02 ± 0.58	1.10 ± 0.34^*^	3.12 ± 0.92^*^	0.51 ± 0.15^*^	330 ± 123^*^	2.58 ± 0.33
Mut-8	0.86 ± 0.20	0.55 ± 0.18	1.41 ± 0.38	0.29 ± 0.04	190 ± 41	1.98 ± 0.53
Mut-9	0.81 ± 0.07	0.47 ± 0.04	1.28 ± 0.11	0.23 ± 0.03	143 ± 37	2.36 ± 0.32

Isolated strains were scaled up to 25 mL culture volumes and harvested after 4 days. Chlorophyll (Chl) a, Chl b, total Chl and total carotenoid (Car) measurements were estimated using a spectrophotometer [40]. Lutein content was measured by high-performance liquid chromatography against a lutein standard. DCW, dry cell weight. Data represent mean values (*n* = 3, except for Mut-6 for which *n* = 2) ± the standard deviation from the mean. Multiple comparisons were performed using one-way ANOVA, with a Bonferroni post hoc test, to compare each mutant to the CC-125 parental strain (*p < 0.05; **p < 0.01; ***p < 0.001; ****p < 0.0001)

### Label-free quantitative (LFQ) proteomics comparing Mut-5 and parental strain CC-125

The lutein and total Chl contents of Mut-5 were 5.4-fold and 3.0-fold higher than the CC-125 control strain, respectively (Table 1). This presents us with several questions: where is Mut-5 storing its extra pigments, and what other metabolic processes could be involved in its increased pigment accumulation? Moreover, how did Mut-5 simultaneously produce more Chl a, whose excitation produces detrimental singlet oxygen radicals, while exhibiting better survival under norflurazon and high light stress during mutant selection? To answer these questions, LFQ proteomics was performed to compare differentially abundant proteins between Mut-5 and CC-125 strains. Given increasing evidence for the translational regulation of photosynthesis-related proteins in *C. reinhardtii* [41–43], a comparative proteomics approach was implemented to reveal metabolic changes that may not be ascertained using transcriptomics.

#### Time-point selection for comparative proteomics analysis

**Fig. 1:**
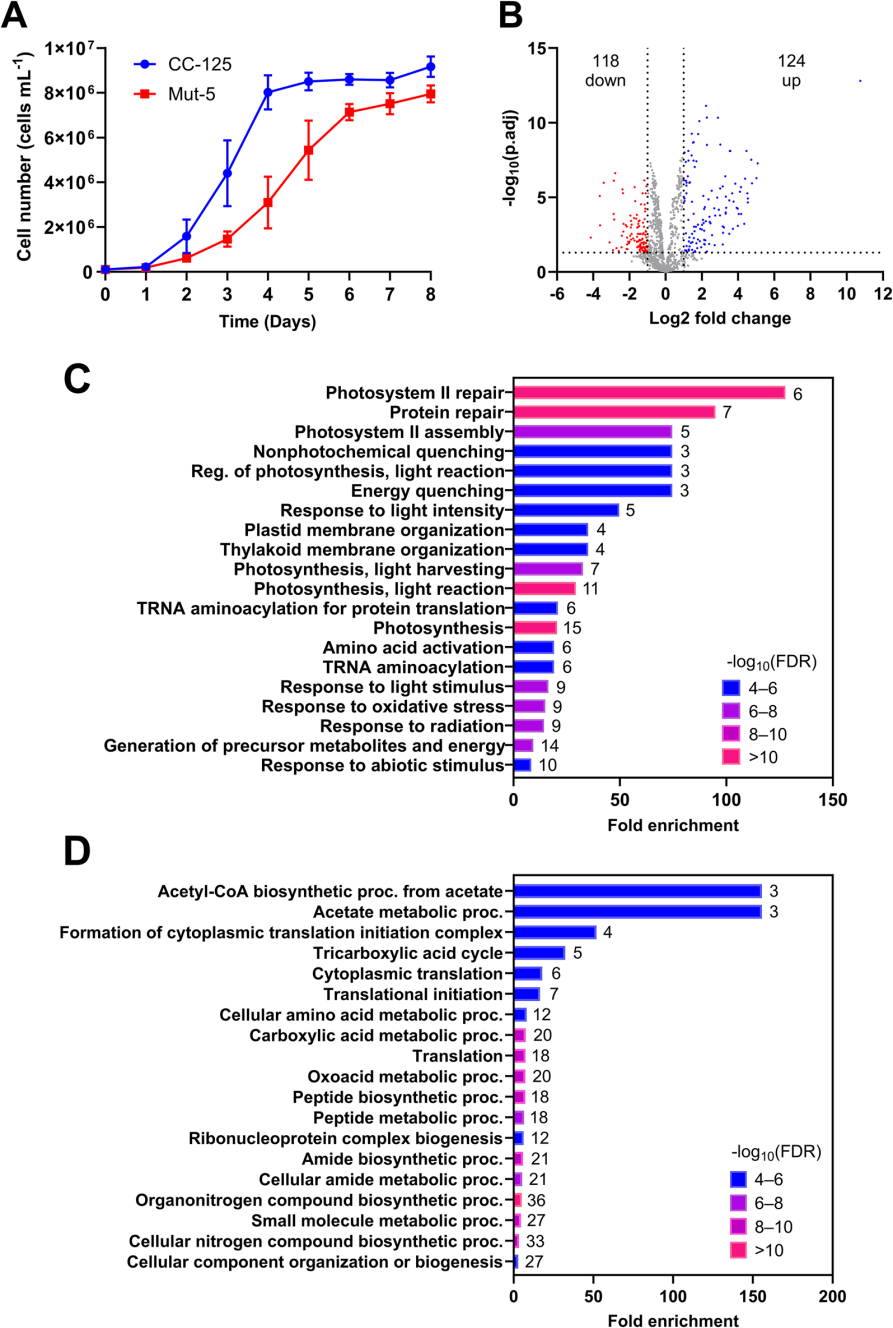
Proteomics data overview. **A** Growth of CC-125 and Mut-5 cultivated under standard conditions to determine time-point selection for comparative proteomics. Data points represent the mean of three independent replicates; error bars indicate the standard deviation from the mean. **B** Volcano plot showing ﻿−Log_10_-transformed adjusted *p*-values against the Log_2_ fold changes for reproducibly quantifiable proteins. Log_2_ fold change is presented as Mut-5/CC-125. Data points coloured blue and red represent proteins of increased and decreased abundance in Mut-5, respectively. Dotted lines represent the cut-offs for significantly differential protein abundance between Mut-5 and CC-125 (adjusted *p*-value < 0.05; Log_2_ fold change < -1 or < 1). **C** Bar plot showing the top 20 biological process gene ontology (GO) terms enriched in proteins more abundant in Mut-5 compared to CC-125. The number of significantly upregulated proteins associated with each biological process GO term is indicated to the right of each bar. −Log_10_-transformed false discovery rate is indicated by colour. **D** Bar plot showing the top 20 biological process gene ontology terms enriched in proteins less abundant in Mut-5 compared to CC-125. The number of significantly downregulated proteins associated with each biological process GO term is indicated to the right of each bar. ﻿−Log_10_-transformed false discovery rate is indicated by colour

The control strain CC-125 was harvested at Day 4, and Mut-5 harvested at Day 6 for comparing the proteomes. These time points represent late-log/early-stationary phase for both strains, with no significant difference in cells mL^−1^, as determined during an 8 day growth study (Fig. 1A). The specific growth rate of Mut-5 (0.027 ± 0.004 h^−1^) was lower than that of CC-125 (0.039 ± 0.009 h^−1^); although this was not statistically significant (*p* = 0.121; Student’s *t*-test), the lack of significance was due to variance between replicates. The total carotenoids and Chl per cell for both strains remained relatively stable across all time points and were consistently higher in Mut-5 (Additional File 2), suggesting that proteins involved in pigment production and/or storage would continue to be stably expressed in Mut-5 at the time points selected.

#### Proteomics overview

Proteins were extracted from CC-125 and Mut-5 cultures in biological triplicate and analysed by comparative shotgun LFQ proteomics. Of the 1876 proteins identified using Maxquant MaxLFQ analysis [44], 1075 proteins were quantified using LFQ Analyst [45] and compared between samples. Proteins with Mut-5/CC-125 Log_2_ fold changes (Log_2_FC) > 1 and adjusted *p*-values < 0.05 were considered to have significantly differential expression between strains; 242 (22.5%) of proteins differed significantly between Mut-5 and CC-125, of which 124 were upregulated and 118 were downregulated in Mut-5 compared to CC-125 (Fig. 1B). For a list of all detected and quantified proteins, refer to Additional File 4.

Functional enrichment analysis of the differentially enriched proteins was performed using ShinyGO [46]. Gene ontology (GO) biological process (BP) terms including PSII repair and assembly, non-photochemical quenching (NPQ), and thylakoid membrane organization were enriched in Mut-5 compared to CC-125, suggestive of constitutive activation of high light stress responses in Mut-5 (Fig. 1C). GO-BP terms related to acetate and the tricarboxylic acid (TCA) cycle were significantly enriched in proteins downregulated in Mut-5 (Fig. 1D), while translation initiation, amino acid metabolism, and peptide biosynthesis were also downregulated; this combination of decreased translation and carbon metabolism may explain the extended lag phase and slower growth of Mut-5 (Fig. 1A).

#### Pigment binding and carrier proteins are more abundant in Mut-5

**Fig. 2:**
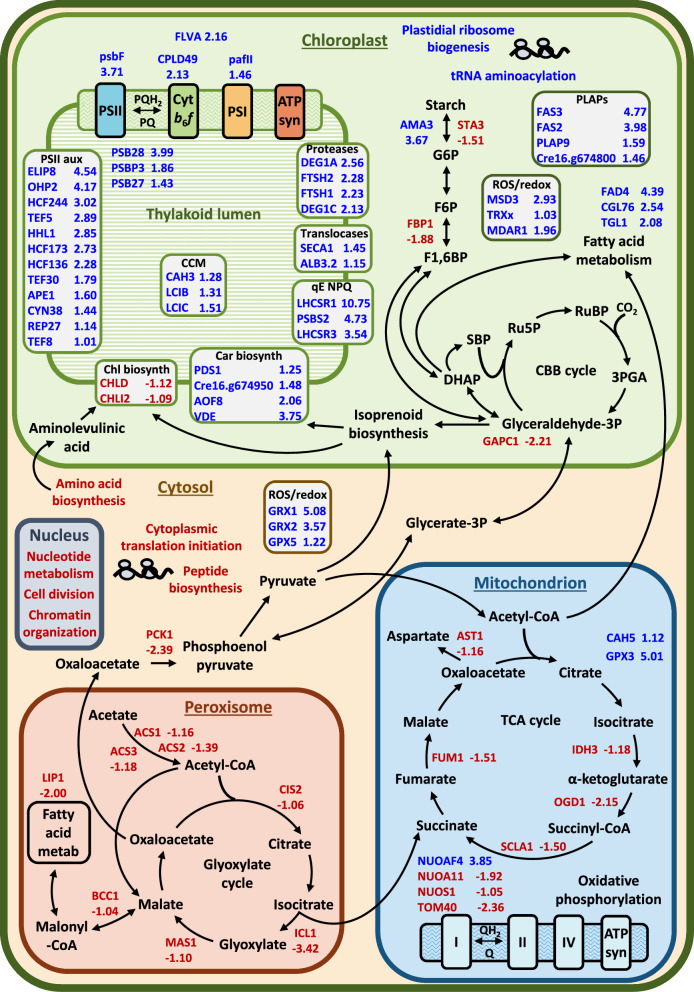
Schematic representation of proteins and pathways differentially enriched in Mut-5 vs CC-125. 73/242 proteins with significantly higher (blue) or lower (red) abundance in Mut-5 vs CC-125 are shown (Log2 fold change > 1 or < 1; adjusted *p*-value < 0.05). Pathways coloured blue/red were significantly more/less enriched in Mut-5 vs CC-125, respectively. Values indicate the mean (*n* = 3) Mut-5/CC-125 Log_2_-transformed fold change for each protein. PSII/PSI, photosystem II/I; Cyt *b*_6_*f*, cytochrome *b*_6_*f*; ATP syn, ATP synthase; (P)Q, (plasta)quinone; PSII aux, PSII auxiliary proteins; qE NPQ, energy-dependent non-photochemical quenching; CCM, carbon-concentrating mechanism; Chl biosynth, chlorophyll biosynthesis; Car biosynth, carotenoid biosynthesis; FLVA, Flavodiiron protein-A; psbF/PSBP3/PSB27/PSB28/PSBS2, PSII subunits; CPLD49, Conserved in the Plant Lineage and Diatoms-49; pafII, PSI assembly factor-II; ELIP8, early light-inducible protein-8; OHP2, ONE-HELIX PROTEIN-2; HCF244/173/136, HIGH CHLOROPHYLL FLUORESCENCE-244/173/136; TEF5/30/8, thylakoid lumenal protein-5/30/8; HHL1, hypersensitive to high light-1; APE1, acclimation of photosynthesis to environment 1; CYN38, cyclophilin-38; REP27, repair protein 27; DEG1A/1C, degradation of periplasmic proteins protease-1A/1C; FTSH1/2, filamentation temperature-sensitive-1/2; SECA1, sorting factor-A1; ALB3.2, ALBINO3-like translocon protein-3.2; LHCSR1/3, light-harvesting complex stress-related protein-1/3; CAH3/5, carbonic anhydrase-3/5; PDS1, phytoene desaturase-1; AOF8, flavin-containing amino oxidase-8; VDE, violaxanthin de-epoxidase; CHLD/I2, magnesium-protoporphyrin IX chelatase subunit D/I2; PLAP(s), plastid lipid-associated protein(s); FAS2/3, fasciclin-like protein-2/3; AMA3, alpha-amylase-3; STA3, soluble starch synthase III; G6P, glucose 6-phosphate; F6P, fructose 6-phosphate; F1,6BP, Fructose-1,6-bisphosphate; FBP1, Fructose-1,6-bisphosphatase-1; DHAP, Dihydroxyacetone phosphate; SBP, sedoheptulose-1,7-bisphosphate; Ru5P, ribulose 5-phosphate; RuBP, Ribulose-1,5-bisphosphate; 3PGA, 3-phosphoglycerate; GAPC1, chloroplastic glyceraldehyde-3-phosphate dehydrogenase-1; FAD4, fatty acid desaturase-4; CGL76; conserved in the green lineage-76; TGL1, triacylglycerol lipase-1; MSD3, manganese superoxide dismutase-3; TRXx, thioredoxin x; MDAR1, monodehydroascorbate reductase; GRX1/2, glutaredoxin-1/2; GPX5/3, glutathione peroxidase-1/3; AST1, Aspartate aminotransferase; IDH3, Isocitrate dehydrogenase-3; OGD1, 2-oxoglutarate dehydrogenase subunit-E1; SCLA1, Succinyl-CoA ligase α-chain; FUM1, Fumarate hydratase; NUOAF4/S1, NADH:ubiquinone oxidoreductase subunit AF4/S1; TIM17/22, translocase of the inner membrane-17/22; TOM40, 40 kDa translocon at mitochondrial outer envelope membrane; ACS1/2/3, Acetyl-coenzyme A synthetase-1/2/3; CIS2, citrate synthase-2; ICL1, isocitrate lyase-1; MAS1, malate synthase-1; BCC1, Acetyl-coenzyme A biotin carboxyl carrier; PCK1, Phosphoenolpyruvate carboxykinase-1

**Fig. 3:**
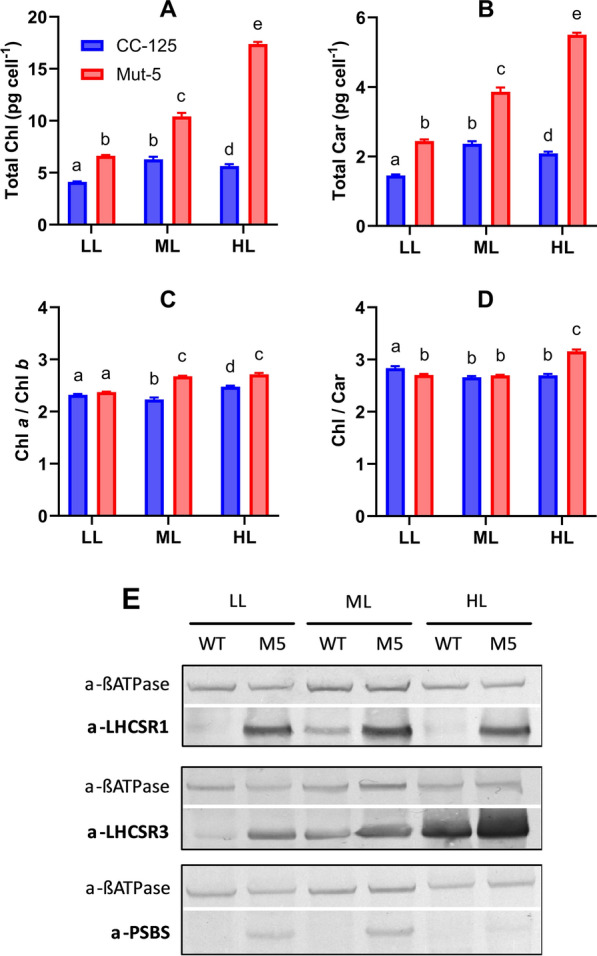
Pigment and qE protein accumulation in CC-125 and Mut-5 cultured in three light conditions. Pigment contents and qE protein accumulation in *C. reinhardtii* strains CC-125 (blue) and Mut-5 (red) acclimated to low light (LL; 70 µmol photons m^2^ s^−1^), medium light (ML; 150 µmol photons m^2^ s^−1^), and high light (HL; 400 µmol photons m^2^ s^−1^). Total chlorophyll (Chl) content in pg per cell **A**, total carotenoid (Car) content in pg per cell **B**, Chl a/Chl b ratios **C**, and Chl/Car ratios **D** are displayed. Data are represented as the mean of 3 independent replicates with error bars depicting standard deviation from the mean. For each parameter, significant differences between each strain and light condition were calculated by two-way ANOVA with a multiple comparisons test (compare cell means regardless of rows and columns) and post hoc Bonferroni correction. Means marked with the same letter are not significantly different (*p*-value > 0.05). **E** Immunoblots showing LHCSR1, LHCSR3, and PSBS expression in CC-125 (WT) and Mut-5 (M5) under LL, ML, and HL. Antibodies detecting α-ATPase β-subunit were included as a control

The greatest increase in protein expression in Mut-5 relative to CC-125 was for light-harvesting complex stress-related protein (LHCSR) 1 (Fig. 2), which had a Log_2_FC of 10.75, translating to a steep linear fold change of 1722. Similarly, LHCSR3 exhibited a Log_2_FC of 3.54. The higher LHCSR1 and LHCSR3 protein levels were later confirmed biochemically for three light conditions via immunoblots as shown in Fig. 3. The LHCSR proteins are key mediators of the energy-dependent (qE) component of NPQ under high light stress [47]. LHCSR1 contains approximately three carotenoid binding sites, two of which have a high affinity for lutein, and an estimated eight Chl binding sites, which preferentially bind Chl a over Chl b [48, 49]. Likewise, ~ eight Chl a molecules and three carotenoids, primarily lutein and violaxanthin, are estimated to occupy each LHCSR3 apoprotein [48, 49]. Two other LHC-like proteins, which contain putative Chl and carotenoid binding sites and are involved in PSII assembly and repair, were significantly enriched in Mut-5: early light-inducible protein (ELIP) 8 and one-helix protein (OHP) 2 (Fig. 2).

Seventeen LHC proteins forming the antenna systems of PSI and PSII (LHCI and LHCII, respectively) which bind Chl a and lutein, were detected by proteomics; although their levels were slightly higher in Mut-5 than in CC-125, they were not statistically significant (Additional File 4). This suggests that the increased *LHC-like* protein expression may be providing a storage sink for the excess lutein and Chl a, rather than the LHC antenna proteins.

Two plastid lipid-associated proteins (PLAPs), PLAP9 and Cre16.g674800, displayed increased abundance in Mut-5 (Fig. 2). PLAPs are associated with plastoglobules and the carotenoid-rich eye-spot in *C. reinhardtii* [50]. In plants, PLAPs have roles in photoprotection and carotenoid storage during chromoplast development and potentially play a similar role in carotenoid sequestration in *C. reinhardtii* [51]. Moreover, two fasciclin-like proteins, FAS3 and FAS2, which are membrane-bound peptides that are also associated with the eye-spot [52], were significantly upregulated in Mut-5 (Fig. 2). The functions of these proteins in *C reinhardtii* are as of yet unknown, but their potential involvement in carotenoid accumulation and storage could be worth further investigation.

#### Carotenoid biosynthesis enzymes are more abundant in Mut-5, while chlorophyll biosynthesis proteins are less abundant

Several proteins involved in carotenoid biosynthesis were more abundant in Mut-5, including a violaxanthin de-epoxidase and phytoene desaturase (Fig. 2). This violaxanthin de-epoxidase is unique to *C. reinhardtii* and is bioinformatically predicted to be involved in lutein and β-carotene biosynthesis, in addition to its role in NPQ via the xanthophyll cycle [53]. Two putative carotenoid biosynthetic enzymes, Cre16.g674950 and Cre13.g587500, also exhibited significantly higher abundance in Mut-5 (Fig. 2), while the comparative levels of zeta-carotene desaturase (Cre07.g314150), prolycopene isomerase (Cre16.g651923), and a flavin amine oxidase (Cre12.g560900) were higher in Mut-5 but not significantly so, according to our relatively stringent Log_2_FC cut-off of 1 (Additional File 4). The increase in carotenoid biosynthetic enzymes begins to explain the higher total carotenoid content of Mut-5.

No Chl biosynthesis enzymes were significantly more abundant in Mut-5, despite its higher Chl content. Conversely, Mg-protoporphyrin chelatase subunits CHLD and CHLI2, which constitute a key enzyme in Chl biosynthesis, were significantly lower in Mut-5 (Fig. 2). Ten other predicted Chl biosynthetic enzymes were detected by proteomics, but their levels were not significantly different to those of CC-125. No Chl catabolic enzymes were detected by proteomics (Additional File 4). The incongruence between the observed increase in Chl content and reduced abundance of Chl biosynthetic enzymes in Mut-5 suggests that the mechanisms driving Chl turnover and/or storage may be disrupted in Mut-5.

#### Photoprotective, reactive oxygen species stress, and redox homeostasis-related proteins are more abundant in Mut-5

In addition to the LHCSR proteins, PSII subunit S protein (PSBS), another protein crucial for qE NPQ, exhibited high relative abundance in Mut-5 (Fig. 2), as also evidenced by immunoblotting using an anti-PSBS serum specific for the *C. reinhardtii* protein isoform [54, 55]. The precise function of PSBS is currently unknown in *C. reinhardtii*, especially since this gene is usually transcriptionally silent under non-stressing light conditions [55, 56], although its expression is necessary for full LHCSR3 accumulation and acclimation to high light [47, 56, 57]. Various uncharacterized proteins that share homology to *Arabidopsis* NPQ-related proteins were more abundant in Mut-5, including CPLD42 (Cre01.g004450; Log_2_FC = 1.26), which shares 40% sequence identity (BLAST E-value 8e-43) with NPQ protein FLUCTUATING-LIGHT-ACCLIMATION PROTEIN1 [58], as well as Cre13.g586050 (Log_2_FC = 3.32) and CGLD13 (Cre03.g181250; Log_2_FC = 1.39), whose amino acid sequences are similar to *Arabidopsis* SUPPRESSOR OF QUENCHING1 (At1g56500) and RELAXATION OF QH1 (At4g31530), respectively (Additional File 4).

As shown in Fig. 1C, PSII repair and PSII assembly were the most highly enriched pathways in Mut-5. However, the core subunits of the PSII reaction centre (RC) did not differ significantly between Mut-5 and CC-125, with the exception of cytochrome *b*_559_ subunit β and the putative oxygen-evolving complex protein PSBP3, which were significantly enhanced in Mut-5 (Fig. 2). Proteins involved in PSII-RC PsbA (D1) protein turnover were enriched in Mut-5 (Fig. 2), including HIGH CHLOROPHYLL FLUORESCENCE (HCF) 244, HCF173, HCF136, and OHP2, which cooperate to stabilize *psbA* mRNA and enhance its translation [59–61], alongside the high light-induced D1 proteases FTSH1, FTSH2, DEG1A, and DEG1C [62, 63]. Notably, levels of D1 were similar between Mut-5 and CC-125 (Log_2_FC = 0.30, adjusted *p*-value = 0.052). Other PSII assembly and repair proteins enriched in Mut-5 include PSB28, whose orthologue in *Synechocystis* plays a role in the biosynthesis of Chl and its incorporation into the PSII-RC [64], and TEF5, a homologue of *Arabidopsis* psb33 that mediates PS-LHCII interactions and energy transfer under high light [65, 66]. Assembly factors for cytochrome *b*_6_*f* (Conserved in the Plant Lineage and Diatoms 49) and PSI (PSI assembly factor-II) were also more abundant in Mut-5 (Fig. 2).

Proteins involved in reactive oxygen species (ROS) stress and redox homeostasis displayed particularly high fold increases in Mut-5 compared to CC-125 (Fig. 2). For example, two known glutaredoxins (glutaredoxins 1 and 2; Fig. 2) and one putative glutaredoxin (cre06.g261500; Log2 = 3.31) were comparatively highly expressed. Two glutathione peroxidases (GPX3 and GPX5; Fig. 2), which are highly induced by singlet oxygen [67], were also upregulated. Other enriched redox-related proteins of note include a chloroplastic manganese superoxide dismutase (Fig. 2), whose expression is triggered by Fe/Mn deficiency or H_2_O_2_ stress [68], thioredoxin x, methionine sulfoxide reductase 1B, and monodehydroascorbate reductase 1 (Fig. 2).

Collectively, the induction of photoprotective and redox homeostasis proteins is indicative of a widespread stress response in Mut-5 and may have contributed to its survival during selection in norflurazon and high light.

#### Central carbon metabolism and respiration are suppressed in Mut-5

As indicated by the gene ontology analysis, acetate metabolism appears to be downregulated in Mut-5 compared to CC-125 (Fig. 1D). Mixotrophic growth using acetate as a carbon source is reliant upon the glyoxylate cycle, and crucially the expression of isocitrate lyase 1 [69], which is strongly downregulated in Mut-5 (Fig. 2). Moreover, three acetyl-CoA synthetases (ACS1, ACS2, and ACS3), which oversee acetate uptake in *C. reinhardtii*, are significantly downregulated in Mut-5, as well as two glyoxylate cycle enzymes, citrate synthase 2 and malate synthase 1, pointing towards a trend of downregulated acetate metabolism in Mut-5 [70](Fig. 2). Further to this, four TCA cycle enzymes were significantly less abundant in Mut-5 compared to CC-125 (Fig. 2), suggesting downregulated central carbon respiration.

Enzymes in the Calvin–Benson–Bentham cycle were overall slightly downregulated in Mut-5, albeit not significantly so (Additional File 4). However, downstream pathways linked to Calvin–Benson–Bentham cycle products were downregulated in Mut-5. Fructose-1,6-bisphosphatase 1, which feeds photosynthetically derived sugars into the gluconeogenesis and starch pathways, was reduced, as well as the chloroplastic glyceraldehyde 3-phosphate dehydrogenase, which is also linked to glycolysis and gluconeogenesis (Fig. 2). Furthermore, increased alpha-amylase 3 and decreased soluble starch synthase III expression in Mut-5 suggests a breakdown of starch (Fig. 2); this, in conjunction with reduced gluconeogenesis, is suggestive of carbon limitation, with Mut-5 inducing glycogen catabolism to compensate for the sugar deficit. Further to this, levels of key proteins involved in the carbon-concentrating mechanism were significantly higher in Mut-5, including two carbonic anhydrases and two low-CO_2_-inducible proteins (Fig. 2), which is indicative of carbon limitation, or at least induction of the carbon limitation response.

Conflicting conclusions can be drawn with regard to fatty acid metabolism in Mut-5. Fatty acid desaturase 4 is strongly upregulated in Mut-5, while diacylglycerol lipase 1 was significantly lower, which is suggestive of fatty acid biosynthesis; this is in contrast with the upregulation of two enzymes with predicted roles in triacylglycerol degradation: conserved in green lineage 76 [71] and the putative triacylglycerol lipase 1 (Fig. 2). While the lack of characterization of these enzymes makes it difficult to discern the direction of lipid metabolism in Mut-5, the combination of starch degradation and general suppression of central carbon metabolism would suggest that lipid beta-oxidation may be upregulated to generate acetyl-CoA, given the apparent suppression of acetate uptake and metabolism.

Two subunits of the mitochondrial oxidative phosphorylation complex I were less abundant in Mut-5, alongside an associated mitochondrial translocase (Fig. 2); the deficit in NADH caused by the downregulation of the TCA cycle likely had a negative effect on oxidative phosphorylation, and thus respiration. The complex I chaperone NUOAF4 was, however, upregulated in Mut-5, which may be related to the general ROS stress response occurring throughout the cell.

#### Mut-5 exhibits differences in translation and transcription factor expression

The predominant differences in regulatory factors were related to cytosolic translation (Additional File 4), which was heavily decreased in Mut-5. Eight subunits of eukaryotic translation initiation factors 2 and 3 were significantly lower in Mut-5 (Table 2). Cytosolic ribosomal subunits and rRNA methylation complex factors were also lower (Additional File 4). This, in combination with reduced amino acid biosynthesis, indicates reduced protein production in the Mut-5 cytosol. Notably, chloroplastic tRNA-aminoacyl synthetase expression and ribosome biogenesis were increased in Mut-5; this increase in ribosome biogenesis and amino acid activation, but not in amino acid biosynthesis, supports the notion of increased protein turnover in the Mut-5 chloroplast.

**Table 2:** Predicted translation and transcription factors differentially abundant in Mut-5

Phytozome ID	Gene name	Log_2_FC	p.adj	Description
*Predicted translation factors*				
Cre03.g165000	EFG10	1.64	4.56E−02	Translation elongation factor EFG/EF2, LepA-related
Cre12.g519180	EFT1a	1.04	9.93E−09	Elongation factor Ts-like protein
Cre06.g298100	SUI1A	− 1.45	7.05E−03	Translation initiation protein
Cre12.g490000	EIF2A	− 1.75	2.47E−04	Eukaryotic translation initiation factor 2 subunit 1
Cre06.g298350	FAP224	− 1.26	2.96E−03	Flagellar-associated protein FAP224
Cre17.g697450	EIF3L	− 1.08	3.73E−02	Eukaryotic translation initiation factor 3 subunit L
Cre03.g190100	EIF3B	− 1.72	7.88E−03	Eukaryotic translation initiation factor 3, subunit B
Cre04.g217550	EIF3C	− 1.59	1.50E−04	Eukaryotic translation initiation factor 3, subunit C
Cre05.g242300	EIF3D	− 1.88	6.77E−04	Eukaryotic translation initiation factor 3, subunit D
Cre16.g676314	EIF3H	− 1.58	4.40E−03	Eukaryotic translation initiation factor 3, subunit H
Cre06.g259150	EFG8	– 1.09	4.68E−02	Elongation factor Tu
Cre09.g415800		− 1.43	1.97E−02	Programmed cell death protein
Cre06.g284750	EFG3	− 1.22	1.41E−02	Translation elongation factor Tu family protein
Cre13.g587050	ERF1	− 1.41	2.04E−02	Eukaryotic release factor 1
*Predicted transcription factors*				
Cre10.g461900		2.93	6.82E−05	Aldo/ keto reductase; homologous to AtbZIP11/ ATB2
Cre06.g275100		− 3.62	7.57E−04	Nucleolin; Splicing Factor 3B, Subunit 4
Cre03.g152150		− 1.13	2.92E−02	C2H2-type domain-containing protein
*Proteasome regulatory subunits*				
Cre17.g710150	RPT4	− 1.95	1.67E−04	26S proteasome regulatory subunit

Given the importance of translational and post-translational regulation in *C. reinhardtii*, it may be fruitful to consider the as-of-yet uncharacterized RNA processing, translation factors, and ubiquitin proteasome components that are differentially regulated in Mut-5, as regulatory factors governing its phenotype may well be among them. Examples include the programmed cell death factor with a gene ontology biological process term associated with negative regulation of transcription and a predicted mRNA-interacting domain, a predicted translation factor FAP244 with a basic leucine zipper domain, translation elongation factor Tu (EFG) 3 and EFG8, as well as a 26S proteasome regulatory subunit RPT4, among others (Table 2; Additional File 4). The expression of eukaryotic release factor 1 was lower in Mut-5; interestingly, its higher plant orthologues (*e.g. Brassica oleracea* Bo8g065090 and Bo9g007980) are suppressed by ORANGE, a carotenoid biosynthesis-inducing regulatory protein also present in *C. reinhardtii* [26, 72]. Upregulated translation factors include EFG10 and elongation factor Ts-like protein (Table 2).

At least three potential transcription factors were differentially enriched in Mut-5 (Table 2), including an uncharacterized aldo/keto reductase Cre10.g461900, which shares homology with the *A. thaliana* protein ATB2 (BLAST E-value 3e-26, 30% identity), a light-regulated bZIP transcription factor [73]. Accumulation of the putative transcription factor nucleolin (Cre06.g275100) was significantly lower in Mut-5 compared to CC-125. Its function is currently unknown, but it is upregulated in low CO_2_ conditions [74, 75] and in *SAK1* mutants, which lack a ROS response [76]. Levels of a predicted C2H2 transcription factor family protein of unknown function (Cre03.g152150) were also significantly lower in Mut-5 (Table 2). However, no known transcriptional activators/repressors involved in the control of photoprotective gene expression were found to be differentially regulated between Mut-5 and the parental strain [77–79].

### LHCSR protein expression and pigment accumulation are higher in Mut-5 than in CC-125 under varying light intensities

The strong constitutive expression of NPQ-related protein expression in Mut-5, particularly LHCSR1 and LHCSR3, suggests that these proteins may in part explain the increased pigment content. The proteomics data also suggest that a high light stress response is triggered in Mut-5. We performed pigment analyses and immunoblots (Fig. 3) in CC-125 and Mut-5 under three light intensities: low light (LL; 70 μmol photons m^2^ s^−1^), medium light (ML; 150 μmol photons m^2^ s^−1^), and high light (HL; 400 μmol photons m^2^ s^−1^). The ML intensity was selected as this reflects that of the standard conditions used for the earlier HPLC and proteomics experiments. This enabled us to (i) understand how pigment accumulation is affected in Mut-5 by altering the light conditions, (ii) explore the relationship between pigment hyperaccumulation and LHCSR protein expression, and (iii) validate the proteomics data with regards to the constitutive expression of NPQ-related proteins.

The total Chl and carotenoid contents were significantly higher in Mut-5 compared to CC-125 across the three light conditions tested (Fig. 3A, B). Interestingly, the total pigments per cell increased in Mut-5 with increasing light intensity; this contrasts with the CC-125 strain, in which the carotenoids and Chl contents were highest at ML, decreasing under HL conditions (Fig. 3A, B). The Mut-5 total Chl content under HL was threefold higher than that of CC-125 under the same light intensity, and 1.7-fold higher than that of Mut-5 grown under ML. The Chl a/Chl b ratio for CC-125 was lowest in ML and highest in HL, indicating a slight reduction in PSII antenna size in response to the higher light intensity (Fig. 3C). However, in Mut-5, the Chl a/Chl b ratio was consistently higher than that of CC-125, increasing under ML conditions and remaining high under HL conditions. These differences in Chl a/Chl b ratio suggest differences in PS structure and antenna size regulation between CC-125 and Mut-5 in the presence of acetate (Fig. 3C). The Chl/carotenoid ratio was higher in CC-125 compared to Mut-5 under LL, and comparable in both strains under ML conditions. Under HL, the Chl/carotenoid ratio was significantly higher in Mut-5 compared to CC-125 (Fig. 3D).

To investigate the involvement of LHCSR proteins in pigment composition, immunodecoration experiments of total algal protein extracts were performed on the same samples examined for pigment composition to assess LHCSR1, LHCSR3, and PSBS protein levels (Fig. 3). Under the three light conditions tested, the immunoblots indicated that LHCSR1 was consistently highly expressed in Mut-5, while being barely detectable in the CC-125 control strain (Fig. 3E). This is especially apparent under ML conditions, thus bolstering the validity of the proteomics dataset, which indicated a very high 10.45-fold increase in LHCSR1 under similar cultivation conditions (Fig. 2). The sustained overexpression of LHCSR1 in Mut-5, even under LL conditions, may indicate disrupted regulation of light-induced responses, or alternatively biochemical mimicry of high light stress conditions, *e.g*. by increased ROS levels, lower thylakoid lumenal pH, or an excessively reduced plastoquinone pool. LHCSR3 accumulation increased with increasing light intensity in both Mut-5 and CC-125, but this effect was more pronounced in Mut-5 (Fig. 3E). The expression of LHCSR3 is also intimately linked to CO_2_ availability; typically, under mixotrophic conditions, LHCSR3 accumulation is limited [80]. The increase in CC-125 LHCSR3 may be due to the increase in photosynthetic activity with increasing light intensity, thus reducing the pool of available CO_2_ and inducing CO_2_-linked LHCSR3 expression. LHCSR3 expression remained relatively high in Mut-5, even in the presence of acetate under LL conditions. PSBS was essentially undetectable in CC-125 under any condition, but bands were visible for all light intensities tested in Mut-5. PSBS expression appeared to be highest under ML conditions in Mut-5, and lowest under HL.

### Mut-5 exhibits high NPQ but lower photosynthetic efficiency

**Fig. 4:**
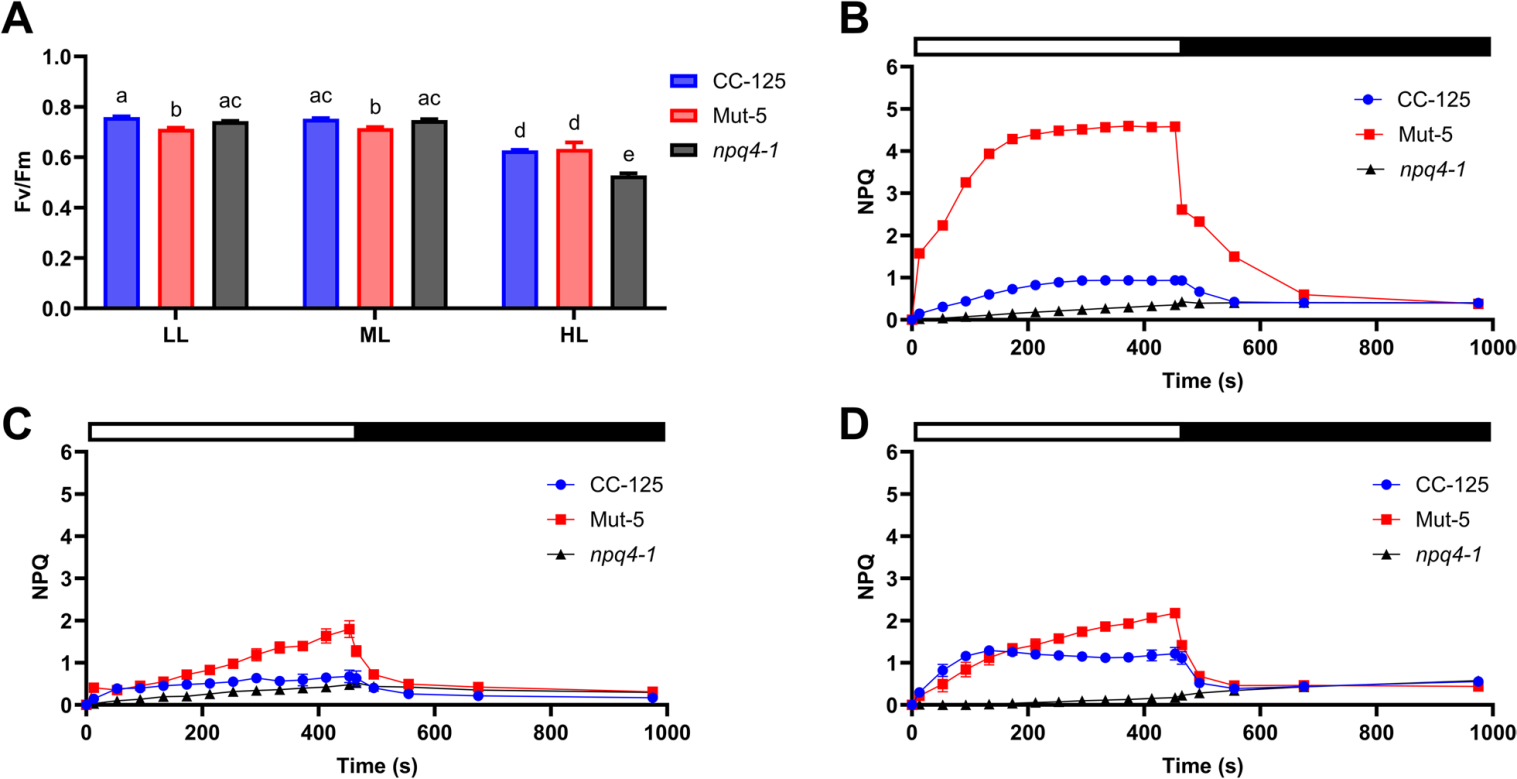
Pulse-amplitude modulation (PAM) fluorescence measurements of *C. reinhardtii* strains CC-125, Mut-5, and *npq4-1* cultured at different light intensities. **A** Maximum photosystem II quantum yield (Fv/Fm) measurements for CC-125, Mut-5, and *npq4-1* acclimated to low light (LL; 70 µmol photons m^2^ s^−1^), medium light (ML; 150 µmol photons m^2^ s^−1^), and high light (HL; 400 µmol photons m^2^ s^−1^) for 8 days. Data are represented as the mean of 3 independent replicates with error bars depicting standard deviation. For each parameter, significant differences between each strain and light condition were calculated by two-way ANOVA with a multiple comparisons test (compare cell means regardless of rows and columns) with post hoc Bonferroni correction. Means marked with the same letter are not significantly different. **B**–**D** Rise and decay kinetics of non-photochemical quenching (NPQ) in CC-125, Mut-5, and *npq4-1* measured following acclimation to **B** LL, **C** ML, and **D** HL conditions. Dark-adapted cells were subjected to illumination with saturating white actinic light (1630 µmol photons m^2^ s^−1^) in intervals for 8 min, followed by relaxation in the dark. White and black bars above the plots represent illuminated and dark periods, respectively

To investigate how the pigment hyperaccumulation and increased qE NPQ proteins influenced photosynthetic efficiency and NPQ in Mut-5 under varying light regimes, we examined NPQ induction and relaxation kinetics. CC-125 and Mut-5 cells were grown under LL, ML, and HL for 8 days in TAP media, the latter condition to ensure maximum expression of the qE NPQ-related proteins in the CC-125 parental strain. The *C. reinhardtii* strain *npq4/lhcsr1*, a mutant lacking both LHCSR1 and LHCSR3 proteins (hereafter referred to as *npq4-1*) and which therefore completely fails to activate the qE NPQ component, was included in the experiment to act as a negative control [81, 82]. After dark adaptation, cells were illuminated with flashes of saturating white actinic light for 8 min to examine NPQ induction and relaxation (Fig. 4). The maximum PSII quantum yield (Fv/Fm) was significantly lower in Mut-5 compared to CC-125 and *npq4-1* under LL and ML, indicating reduced photosynthetic efficiency in Mut-5 at lower light intensities (Fig. 4A); this was also consistent with the slower growth rate (Fig. 1A) and reduced expression of respiration and translation-related proteins (Fig. 2) in Mut-5. At HL, Fv/Fm was similar between Mut-5 and CC-125 (Fig. 4A).

The difference in NPQ between Mut-5 and the control strains acclimated to LL was substantial; by 200 s, the NPQ of Mut-5 was ~ 4.5-fold higher than CC-125 (Fig. 4B). NPQ is typically inactive in low light-acclimated cells and in the presence of acetate [83]; the constitutive expression of LHCSR proteins (Fig. 3E) and suppression of acetate metabolism (Fig. 2) may contribute synchronously to increase the NPQ activity of Mut-5. The extended NPQ relaxation of Mut-5 following initiation of the dark recovery period may be indicative of photoinhibition throughout the 8 min actinic light phase (Fig. 4B). Under ML and HL conditions, NPQ was again highest in Mut-5, but to a lesser extent (Fig. 4C, D). Interestingly, NPQ did not reach a steady-state level in either ML or HL, and instead continued to rise until dark conditions were induced, which may suggest a delayed or cumulative NPQ inductive response in Mut-5.

## Discussion

Here, we generated a lutein hyper-producing mutant of the model green microalga *C. reinhardtii* with constitutively active photoprotection via random mutagenesis, which we characterized using comparative proteomics. Our in-depth study of the Mut-5 proteome enabled us to interrogate the metabolic and regulatory pathways that contribute to its high-pigment and constitutively active photoprotective phenotype, identifying potential metabolic engineering targets for amplifying lutein production in the process.

### Increased LHC-like proteins and PSII protein turnover may contribute to lutein and chlorophyll a sequestration in Mut-5

The increase in Mut-5 carotenoids may be partially attributed to an increase in carotenoid biosynthetic enzymes (Fig. 2). However, the same cannot be said for the increase in Chl a; overall, the abundance of Chl biosynthetic enzymes was reduced in Mut-5 (Fig. 2; Additional File 4). This strongly suggests that the high pigment content of Mut-5 is a result of enhanced pigment storage, as opposed to upregulated biosynthesis. LHC antenna proteins, which are the main Chl binding proteins in Chlorophyta, were slightly enriched in Mut-5, but not significantly so, nor to an extent that might account for the Chl content increase. Furthermore, the Chl a/Chl b ratio of Mut-5 was similar to or higher than that of CC-125, suggesting that the LHCII antenna size was in fact slightly smaller in Mut-5 than in CC-125 (Fig. 3C).

A possible explanation for the enhanced total pigment accumulation could be the enrichment of several LHC-like proteins, namely LHCSR1, LHCSR3, PSBS2, ELIP8, and OHP2 (Fig. 2), which (with the exception of PSBS2) contain numerous predicted or confirmed carotenoid and Chl binding sites [48, 49, 61], implying that these proteins may be acting as pigment storage sinks within the thylakoid membrane. LHCSR1 exhibited a huge Log_2_FC of 10.75, which translates to a linear 1722-fold increase, in Mut-5 compared to CC-125 (Fig. 2). LHCSR1 maintained its comparatively high expression across three light intensities (70, 150, and 400 µmol photons m^2^ s^−1^), which coincided with high total carotenoid and Chl accumulation (Fig. 3). It would therefore be reasonable to credit much of the pigment increase to LHCSR1, which harbours ~ 8 Chl a and 2–4 lutein binding sites [48, 49].

Enrichment of several other LHC-like proteins likely contributed to the increase in pigment biosynthesis and storage capacity. LHC-like proteins contain transmembrane domains and putative pigment binding sites and are associated with thylakoid membranes. In plants, many of these proteins are upregulated following exposure to stress [84]. With the exception of LHCSR1 and LHCSR3, the exact functions of these proteins are currently unclear in *C. reinhardtii*, although their importance in photoprotection, stress, and acclimation processes is becoming increasingly apparent [84, 85]. ELIP8, an orthologue of the Arabidopsis ELIP2, exhibited significantly higher expression in Mut-5 compared to CC-125 (Fig. 2). Arabidopsis ELIP2 binds Chl a and lutein, and its proposed functions include scavenging detached Chls from damaged LHCII proteins, temporarily carrying pigments during LHCII assembly, and negatively modulating Chl biosynthesis [86–90]. Similar observations were made in a time-course transcriptomics study of the lutein-producing alga Desmodesmus sp. JSC3, in which ELIPs were highly expressed during periods of increased lutein accumulation [91]. The PSII biogenesis protein OHP2, which is required for the stability of the PSII reaction centre protein D1 [61], was also highly upregulated in Mut-5 (Fig. 2). OHP2 binds Chl and carotenoid molecules and likely functions as a pigment delivery system for nascent and/or damaged PSII reaction centres in Arabidopsis [92]. In cyanobacteria, OHP orthologues retard Chl degradation from PSII complexes, where they have been proposed to temporarily hold Chl molecules during damaged PSII protein replacement [93]. The LHC-like proteins significantly enriched in Mut-5 may perform similar roles as pigment carriers in *C. reinhardtii*, and are thus likely to be key contributors to the hyperpigmentation phenotype of Mut-5.

Expanding on the potential roles of ELIP8 and OHP2 in pigment sequestration, increased turnover of PSII proteins may also have driven the high pigment levels. High light-induced PSII-associated proteases (FTSH1, FTSH2, Deg1A, and Deg1C) were significantly upregulated in Mut-5 (Fig. 2). At the same time, proteins involved in the synthesis of the PSII-RC D1 protein (HCF244, HCF173, and HCF136) [61] were significantly enriched (Fig. 2). This dual upregulation, which typically occurs during high light stress, may have increased the turnover of D1 and cytochrome *b*_6_*f*, the main targets of the FTSH protease [62, 94]. D1 protein turnover is an important response to high light stress, as the D1 protein is particularly susceptible to photodamage; the removal and replacement of damaged/aberrant D1 is crucial to preventing photoinhibition and further damage from oxygen radicals [95]. Although D1 levels were comparable between Mut-5 and CC-125, D1 turnover may have been higher in Mut-5; a similar phenomenon was observed in a very high light-resistant *C. reinhardtii* strain, which exhibited similar D1 levels to the wild-type strain but increased D1 degradation and synthesis [96]. During the presumably increased PSII-RC degradation and replacement cycle, the pigments initially bound within the RC may be temporarily held by the LHC-like proteins ELIP8 and OHP2, of which there is increased abundance. This may be further promoting Chl and lutein accumulation in the thylakoid. The assumed increased production and degradation of D1 may also be connected to the upregulation of plastidial ribosome biogenesis and tRNA aminoacylation (Additional File 4).

### The regulation of photoprotection is disrupted in Mut-5

Many proteins that are comparatively abundant in Mut-5 (Fig. 2, Additional File 4) are upregulated under high light, ROS stress, or both [97], and many (some overlapping) are regulated by singlet oxygen kinase 1 [76]. High light stress responses in *C. reinhardtii* include activation of NPQ-related gene expression, changes in electron transport and thylakoid membrane ultrastructure, altered stoichiometry of PSI:PSII, synthesis and accumulation of xanthophylls and antioxidants such as tocopherol, and turnover of damaged photosynthetic apparatus components [98]; GO term enrichment (Fig. 1C, D) and individual protein analysis (Fig. 2) revealed involvement of several of these mechanisms in Mut-5. The qE-related protein levels (Fig. 3) and biophysical responses (Fig. 4) were also higher in Mut-5 under three light conditions. Taken together, we can infer that Mut-5 contains a mutation that affects the regulatory pathways governing high light and/ or ROS stress responses, leaving the cell in a perpetually stress-responsive state, even in the absence of cues. This overzealous stress response was likely responsible for the survival of Mut-5 during the norflurazon screening step.

The regulation of *C. reinhardtii* qE-related proteins LHCSR1, LHCSR3, and PSBS, which are notably increased in Mut-5 (Fig. 3E), has recently been a topic of avid investigation in *C. reinhardtii* photosynthesis research [80, 99–102]. Under ambient conditions, the LHCSR and PSBS proteins are virtually undetectable [103]. Previous studies show that these proteins are co-regulated (except in altered CO_2_ conditions [80, 104]), and their expression increases under high light, blue light, and UV irradiation [47, 105, 106]. In a previous study, a *C. reinhardtii* mutant overexpressing LHCSR1 and PSBS (similarly to Mut-5) was shown to retain a missense mutation in a component of a SPA1-COP1 E3 ubiquitin ligase complex, which suppresses qE protein expression [101]. Similarly, another ubiquitin ligase complex CUL4-DDB1DET1 was found to suppress the induction of LHCSR and PSBS proteins [99]. This suggests that part of an E3 ubiquitin ligase complex may have been disrupted in Mut-5, given its similar phenotype. The pigment profiles of these mutants, however, were not reported in these studies [99, 101]. Furthermore, no differential expression was detected for any of the proteins known to be involved in either of the E3 ubiquitin ligase complexes or their transcription factor targets in Mut-5, although a mutation that sterically disrupts complex formation but not protein expression may have arisen, or the regulatory proteins were simply not detected. Many other proteins implicated in gene expression were differentially expressed in Mut-5, most of which were translation factors that present decreased relative abundance compared to CC-125. It is possible that one or more of these regulatory factors contributed to the Mut-5 phenotype, although due to the lack of a detailed genetic analysis of Mut-5, the potential role of these factors could be neither confirmed nor ruled out in our mutant at present. An alternative explanation for the induction of high light and ROS stress responses that should be considered is that a mutation may have caused an increase in intracellular ROS levels.

### Metabolic engineering targets for enhanced lutein accumulation

Although the precise causative mutation(s) of Mut-5 phenotype(s) were not identified, some potential leads for targeted metabolic engineering for enhanced lutein production can still be inferred from our study. For example, the vastly increased expression of LHCSR proteins in Mut-5 suggests that their overexpression could increase lutein storage in *C. reinhardtii*, which could be achieved by knocking out components of the CUL4-DDB1DET1 or COP1-SPA1 complexes [99, 101]. Overexpressing other LHC-like proteins, such as OHP2 and ELIP8, may also enhance carotenoid storage within the thylakoid. Another interesting lead for metabolic engineering is the PAP-fibrillin domain proteins. As discussed above, PAP-fibrillin domain proteins are localized to plastoglobules in plants and perform roles in lipid and carotenoid storage [107, 108]. Interestingly, three PAP-fibrillin proteins were upregulated in Mut-5 (Fig. 2). Recently, a plastoglobule-associated PAP-fibrillin protein was overexpressed in the diatom *Phaeodactylum tricornutum*, leading to a 51% increase in production of the carotenoid fucoxanthin [109]. Functionally characterizing PLAPs in *C. reinhardtii* could be worthwhile, as they could potentially act as a metabolic sink for carotenoids, preventing metabolite-induced feedback inhibition. Moreover, exploring the functions of the transcription and translation factors that were significantly differentially abundant in Mut-5 could reveal unknown regulators of pigment biosynthesis and more widespread photoprotective responses. These targets may additionally be of interest to researchers aiming to increase the production of other xanthophylls such as astaxanthin in green algae. Increasing astaxanthin levels can cause reductions in the number of LHCs and total carotenoids per cell [110–112]; therefore, our targets may offer a means to boost the accumulation capacity of valuable carotenoids.

Given the pervasive effects of the random EMS chemical mutagenesis in Mut-5, which creates an array of single nucleotide polymorphisms scattered across the genome, it may be difficult to pinpoint the genetic occurrences that confer its altered phenotype. Multiple loci are likely to have been affected by the EMS mutagenesis, as was found in previous microalgal EMS mutant generation studies [110, 113, 114], and determining the mutations governing the phenotype(s) of Mut-5 will require repeated genetic back-crosses, followed by phenotype segregation analyses and whole genome sequencing of daughter cells. Hence, these will be the next steps taken towards identifying the causative genetic lesion(s) contained in the genome of Mut-5.

### Increased pigments and photoprotection may come at the expense of slower growth and reduced photosynthetic efficiency

Mut-5 displayed a superior ability to accumulate lutein (5.4-fold) compared to the parental strain (Table 1); however, it also exhibited a reduced growth rate (Fig. 1A), although not statistically significant, and lower maximum quantum photosynthetic yield (Fig. 4A) compared to CC-125. Several factors may have contributed to the reduction in Mut-5 growth, including the heightened Chl accumulation. Lutein is predominantly bound within Chl-binding LHCs in *C. reinhardtii* [14], and Chl levels often correlate with carotenoid content in microalgae [115–117]; therefore, Chl was used as a selection criterion for isolating potential high-lutein mutants during the initial round of mutant selection (Additional File 1). However, high Chl contents also reduce the amount of light that can penetrate algal cultures, reducing light use efficiency, productivity, and biomass accumulation [118, 119]. Although the abundance of Chl biosynthetic enzymes was comparatively lower in Mut-5, total Chl accumulation was higher, even at high light intensities (Fig. 3A). Additionally, the increased NPQ of Mut-5 may compete energetically with productive photochemistry, channelling a disproportionate amount of light energy towards heat dissipation (Fig. 4). These challenges, however, may be a small trade-off for large increases in lutein production, if overall productivity outweighs the growth effects. Moreover, reducing the photosynthetic antenna size by targeting processes responsible for pigment–protein complex accumulation could enhance sunlight-to-biomass conversion efficiency without completely removing lutein-binding LHC proteins [120]. This could be achieved, for example, by suppressing LHC protein translation or by modulating chlorophyll biosynthesis using RNA interference-based silencing approaches [121, 122].

## Conclusions

Using a random mutagenesis, selection, and quantitative proteomics approach, we discovered new targets for increasing pigments and photoprotection in the model green alga *C. reinhardtii*. This offers a series of candidate factors that can be exploited to fully realize the potential of microalgae to replace both wild-type and engineered heterotrophic microorganisms for the industrial production of high-value carotenoids [123–125]. We have demonstrated that it is physiologically possible to increase the number of LHC-like proteins in green algae, which likely increases the number of available carotenoid binding sites, expanding the cellular carotenoid storage capacity of the photosynthetic membranes and enhancing photoprotective properties. Further investigation into the specific causative mutations conferring the photoprotective and pigment-producing phenotypes of Mut-5 could reveal novel regulatory factors involved in these processes.

## Materials and methods

### *C. reinhardtii* cultivation and mutagenesis

The *C. reinhardtii* strain CC-125 was used as the parental strain for mutagenesis. Under standard conditions, cultures were grown mixotrophically in tris–acetate-phosphate (TAP) medium at 25 °C with continuous illumination at 150 μmol photons m^2^ s^−1^ on an orbital shaker set to 120 rpm. Growth was monitored by measuring cell number with a Neubauer cell-counting chamber (Sigma-Aldrich, St. Louis, MO, US) or using a Countess II FL Automated Cell Counter (ThermoFisher, Waltham, MA, US).

To perform chemical mutagenesis, *C. reinhardtii* strain CC-125 was cultured to early exponential phase (1–3 × 10^6^ cells mL^−1^) and harvested by centrifugation at 2000 × *g*, 5 min. Cultures were concentrated tenfold in 0.1 M phosphate buffer (pH 6.8), and EMS was added to a final concentration of 0.3 M. Cells were incubated for 2 h while shaking, after which the EMS mutagenesis reaction was stopped by adding 10 mL sterile 5% sodium thiosulphate (w/v), followed by vortexing and centrifugation (same settings). The pellet was washed with 5% sodium thiosulphate (*w*/*v*), followed by washes with 0.1 M phosphate buffer (pH 6.8) and TAP, and lastly resuspended in TAP. All supernatants following EMS treatment were discarded in a beaker containing sodium thiosulphate crystals. For selection, ~ 1 × 10^6^ cells were spread on to TAP-agar plates supplemented with 0.5, 1, or 3 µM norflurazon and grown inside a high light box with constant illumination at 1050 ± 150 μmol photons m^2^ s^−1^. The 648 colonies that survived the combined pressures of norflurazon and high light were transferred to liquid culture on 96-well plates supplemented with 1 µM norflurazon and grown for 5 days within the high light box. One hundred and forty-four strains exhibiting high specific growth rates and/or high Chl a fluorescence, with ‘high’ defined as one standard deviation above the average growth rate/Chl fluorescence intensity of each individual 96-well plate (Additional File 1), were transferred to 24-well plates and grown under standard conditions, after which their relative pigment contents were estimated using a fluorescence plate reader (Additional File 1).

### Pigment extraction and analysis

To analyse and compare the pigment compositions of Mut-1–9 and CC-125 (Table 1), 4 mL of each 25 mL culture was harvested in triplicate after 96 h of growth under standard conditions by centrifugation at 2000 × *g*, 5 min, 4 °C; pellets were frozen at − 20 °C. All of the following steps were completed in the dark with samples kept on ice. Pellets were resuspended in 1 mL cold 100% acetone, mixed with 425–600 µm diameter glass beads, and incubated on ice for 15 min. Samples were vortexed for 2 min, then incubated on ice for 2 min, for a total of five times, followed by centrifugation at 10,000 × *g*, 5 min, 4 °C. The green supernatant was frozen at − 80 °C prior to analysis. Total Chl and total carotenoid contents were estimated using extinction coefficients as described previously [40]. HPLC was performed on a Dionex UltiMate 3000 HPLC machine with a Hyperselect C_18_ reverse phase column (125 Å pore size, 5 μm particle size, 250 × 4.6 mm), using a previously described method [126]. The separation programme, in which solvent A was ethyl acetate and solvent B was acetonitrile:water 9:1 (*v*/*v*), was as follows: 0–16 min, gradient from 0–60% solvent A; 16–28 min, 60% solvent A. Injection volume was 10 μL, and flow rate set to 1.0 mL min^−1^. Carotenoids were detected at 450 nm absorbance wavelength. Lutein analytical standard (Sigma) was suspended in 100% acetone. Pigment concentrations were determined by interpolating to standard curves generated with known pigment concentrations and normalizing to cell number. For the latter pigment analyses (Fig. 3), pigments were extracted from algal cells using 85% acetone buffered with Na_2_CO_3_ as previously described [127]. Absorption spectra were recorded at room temperature using an Aminco DW-2000 spectrophotometer. Quantification of cellular pigment content, Chl a/b ratio, and Chl/Car ratio were calculated from the deconvoluted spectra of five biological replicates following an established method [128].

### Protein extraction for label-free quantitative (LFQ) proteomics

Twenty mL of late-exponential phase cultures were harvested by centrifugation (2000 × *g*, 18 °C, 5 min) and pellets frozen at − 20 °C. Samples were thawed and resuspended in 1 mL lysis buffer (2% SDS [*w*/*v*], 40 mM Tris base, 60 mM dithiothreitol) with 10 µL Halt^™^ protease inhibitor cocktail (Thermo), frozen at -80 °C for > 24 h, then thawed at 37 °C for 2 min. Each sample was vortexed with 425–600 µM acid-washed glass beads at high speed for 30 s and cooled on ice for 30 s for 10 cycles. Lysed samples were centrifuged at 18,000 ×*g*, 4 °C, 5 min, and stored at − 20 °C. 100 µL of each lysate was purified using a protein 2-D Clean-Up Kit (GE Healthcare, Chicago, IL, US) following the manufacturer’s instructions.

### In-solution protein reduction, alkylation, and digestion for LFQ proteomics

Pellets from the 2-D protein clean-up were resuspended in 50 µL urea buffer (8 M urea; 100 mM Tris–HCl [pH 8.5]; 5 mM dithiothreitol) and sonicated into suspension. Protein concentration was estimated using a NanoDrop^™^ 2000 spectrophotometer (ThermoFisher), and ~ 50 µg protein was transferred to a fresh 1.5 mL protein LoBind Eppendorf tube. Protein samples were reduced by diluting samples up to 10 µL with urea buffer and incubating at 37 °C for 30 min. Proteins were S-alkylated by adding 1 µL 100 mM iodoacetamide and incubating in the dark at room temperature for 30 min. Two micrograms of trypsin/LysC enzyme mix (Promega, Madison, WI, US) was added to the protein solution and incubated at 37 °C for 3 h for LysC digestion, after which the solution was diluted with 75 µL 50 mM Tris–HCl (pH 8.5)/10 mM CaCl_2_ and incubated overnight for trypsin digestion. The digestion was stopped by acidification with 0.05 volumes of 10% trifluoroacetic acid. To desalt the samples, Pierce^®^ C18 spin columns (ThermoFisher) were used according to the manufacturer’s instructions, achieving a peptide yield of ~ 30 µg. Samples were dried using a vacuum evaporator and stored at − 80 °C. Method was adapted from a previous study [129].

### LC–MS/MS and data analysis

Peptide sample pellets were thawed and resuspended in 15 µL loading buffer (97% acetonitrile, 3% H_2_O, 0.1% trifluoroacetic acid [*v*/*v*]). Following 5 min centrifugation (room temperature, max speed), 500 ng of sample was then analysed using a nanoflow LC (Dionex UltiMate 3000 RSLCnano system) coupled online to a Q Exactive HF mass spectrometer (ThermoFisher). Two technical replicates were analysed per biological replicate.

Raw MS data files were processed using MaxQuant version 1.5.2.8 software [44], using the MaxLFQ function. Data were searched against the *C. reinhardtii* proteome (UniprotKB proteome ID UP000006906, last modified December 2019; 18,829 proteins). MaxLFQ parameters were set accordingly: fixed modifications: carbamidomethyl; variable modifications: acetyl (Protein N-term), oxidation; decoy mode: revert; peptide spectrum matches, protein, and site false discovery rates (FDRs): 0.01; special amino acids: arginine and lysine; MS/ MS tolerance (Ion trap MS): 0.5 Daltons (Da); MS/MS tolerance (Fourier transform MS): 20 ppm; MS/MS tolerance (time of flight): 0.5 Da; minimum peptide length: 7; minimum score for modified peptides: 40; peptides used for protein quantification: razor; minimum peptides: 1; minimum razor peptides: 1; minimum unique peptides: 0; minimum ratio count: 2.

The raw MS data files were processed using the label-free quantification option in MaxQuant software (MaxLFQ), which matched MS peaks to peptides in the *C. reinhardtii* proteome using the Andromeda search engine, ten calculated LFQ intensities for identified peptides [130]. As part of the MaxLFQ analysis, common contaminants and non-unique/ razor peptides were filtered out. Matched and quantified proteins were statistically analysed using LFQ Analyst [45] with the following parameters: adjusted *p*-value cut-off, 0.05; Log_2_ fold change cut-off, 1; Perseus-type imputation; Benjamini Hochberg-type FDR correction. GO analysis was performed using ShinyGO [46] with the following parameters: FDR cut-off = 0.05; # pathways to show = 20; and removed redundancy selected. Proteins were annotated and classified using Mercator4 [131], then refined manually using Phytozome 13 [132] and Uniprot [133].

### Immunoblotting of total algal protein extracts

For immunodecoration experiments, total cellular extracts were obtained using a previously described protocol starting from approximately 3 × 10^7^ pelleted cells [134]. Approximately 5 µg of proteins were separated via SDS-PAGE and transferred to a nitrocellulose membrane, and subsequently probed using the following primary antibodies α-LHCSR1 (AS142819, Agrisera, Vännäs, Sweden) α-LHCSR3 (AS142766), and α-ATPase β-subunit (AS05085), as well as the previously described [55] primary α-PSBS antibody, which was kindly provided by Prof. Stefano Caffarri (Aix-Marseille Universitè, France). Blots were developed using an anti-rabbit secondary antibody conjugated to alkaline phosphatase.

### Chlorophyll fluorescence analysis

Chl fluorescence analyses were performed using a Dual PAM 101 fluorometer (Walz, Effeltrich, Germany). Cells were acclimated for seven days under LL, ML, and HL regimes until stationary phase was reached. Measurements were performed on dark-adapted cells kept under agitation on a rotary shaker for > 30 min prior to the analysis. Two millilitres of cells at 1 × 10^8^ cells mL^−1^ was used for measuring standard photosynthetic parameters and NPQ induction curves using an established protocol [127]. Dark-adapted algal cells were exposed to a saturating actinic light pulse (1630 µmol photons m^2^ s^−1^) to determine maximum (*F*_m_) and minimum (*F*_o_) fluorescence emission. During the light phase of NPQ activation and in the dark relaxation phase, each lasting 8 min, saturating light pulses were applied to monitor NPQ. Far red light was used throughout the entire light treatment interval to oxidize the electron transport chain and thus to maximize the contribution of NPQ activation to PSII fluorescence quenching. Maximum PSII quantum yield was calculated using the equation (*F*_m_−*F*_o_)/*F*_m_. NPQ was calculated as previously described [135].

### Statistical analyses

Statistical analyses were performed by one- or two-way ANOVA with Bonferroni post hoc tests using GraphPad Prism software. Error bars represent standard deviation. For one-way ANOVA, statistical significance is represented by asterisks (*p < 0.05, **p < 0.01, ***p < 0.001, ****p < 0.0001. For two-way ANOVA, significance is represented by letters, whereby each letter represents statistical similarity.

### Supplementary Information

**Additional file 1: **Lutein hyperaccumulating mutant screening and selection workflow. **A** Decision tree for initial round of mutant screening. SGR, specific growth rate; mean, average value for microplate on which mutant was grown; SD, standard deviation; ChlFlu, chlorophyll fluorescence; Y, yes; N, no. Strains that fit the criteria for the green boxes were sub-cultured into 24-well plates for the second round of screening. **B** Normalized chlorophyll fluorescence of mutant strains grown for first round of selection. Chlorophyll fluorescence measurements taken by plate reader after 4 days’ growth with the following parameters: excitation 440 nm, emission 680 nm, gains 50. Chlorophyll fluorescence readings for each mutant were normalized to 1/average fluorescence reading for its respective microplate. Each black dot represents an individual mutant strain in an individual well of a 96-well plate. Red line shows the average chlorophyll fluorescence normalized to 1; green line shows the average chlorophyll fluorescence for each plate + 1 standard deviation. **B** Specific growth rates of mutants grown for first round of selection. Specific growth rates were calculated from chlorophyll fluorescence measurements taken by plate reader for each well between Days 1 and 2. Each black dot represents an individual mutant strain in an individual well of a 96-well plate. Green line represents the average mutant growth rate per plate + 1 standard deviation. **D** Total carotenoid (Cars) content of 144 mutant C. reinhardtii strains adjusted to OD750. Total carotenoids were calculated following pigment extraction in pure acetone and subsequent spectrophotometer analysis [40]. Total carotenoid contents were adjusted to cell density at OD750. Each circle represents an individual mutant strain. Red line shows average total carotenoid value/ OD750 for control strain CC-125.

**Additional file 2: **Pigment and growth data for proteomics time-point selection and cultures. **A** Total carotenoid (Cars) and C total chlorophyll (Chl) contents were measured daily for CC-125 and Mut-5 grown under standard conditions on Days 3–8, and are expressed here in pg per cell. Pigment concentrations were estimated using previously described extinction coefficients following acetone extraction and spectrophotometer analysis [40]. **B** Growth curves of samples harvested for proteomics analysis. **D **Final cell density measurements (in cells per mL) for the CC-125 and Mut-5 cultures harvested for proteomics analysis, between which there was no significant difference (*p* = 0.1888; Student’s t-test).

**Additional file 3: **Principle component analysis of the Mut-5 vs CC-125 comparative proteomics data. Principle component analysis (PCA) plot showing six technical replicates of both CC-125 (blue) and Mut-5 (orange). The two strains cluster separately along the PC1 axis, indicative of proteomic differences between the two strains.

**Additional file 4: **Differential abundance analysis and functional annotation of Mut-5 vs CC-125 proteomics data. Table S1 List of all proteins quantified with LFQ Analyst, including GO and MapMan annotations. Table S2 Proteins with significantly higher abundance in Mut-5 compared to CC-125 grouped according to functional annotation. Table S2 Proteins with significantly lower abundance in Mut-5 compared to CC-125 grouped according to functional annotation.

## Data Availability

The datasets used and analysed during the current study are available from the corresponding author on reasonable request.

## Abbreviations

Chl: Chlorophyll
CO_2_: Carbon dioxide
DCW: Dry cell weight
ELIP: Early light-inducible protein
EMS: Ethyl methanesulfonate
F_v_/F_m_: Maximum quantum yield of photosystem II
HL: High light
HPLC: High-performance liquid chromatography
LFQ: Label-free quantitative
LHC: Light-harvesting complex
LHCSR: Light-harvesting stress-related protein
LL: Low light
Log_2_FC: Log_2_ fold change Mut-5/CC-125
ML: Medium light
NPQ: Non-photochemical quenching
OHP: One-helix protein
PLAP(s): Plastid lipid-associated protein(s)
PSBS: Photosystem II subunit S protein
PSII: Photosystem II
qE: Energy-dependent non-photochemical quenching
RC: Reaction centre
ROS: Reactive oxygen species
TAP: Tris-acetate-phosphate
TCA: Tricarboxylic acid cycle
