# Supplementary figures and images for: Proteomic characterization of a lutein-hyperaccumulating Chlamydomonas reinhardtii mutant reveals photoprotection-related factors as targets for increasing cellular carotenoid content
Source: Biotechnol Biofuels Bioprod. 2023 Nov 4;16:166. doi: 10.1186/s13068-023-02421-0 (PMC10625216; doi:10.1186/s13068-023-02421-0)

**A**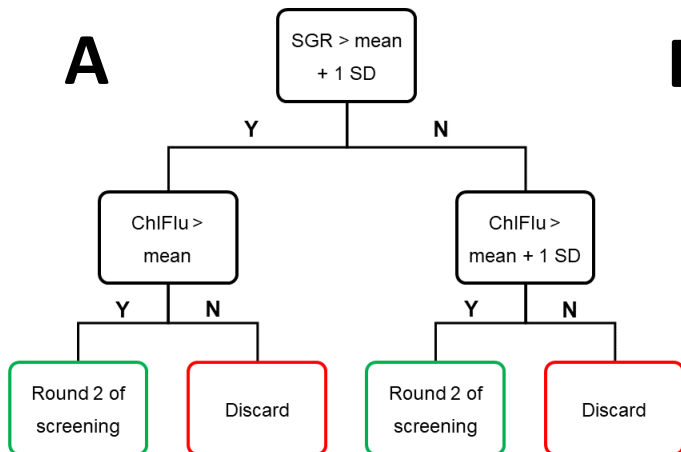**B**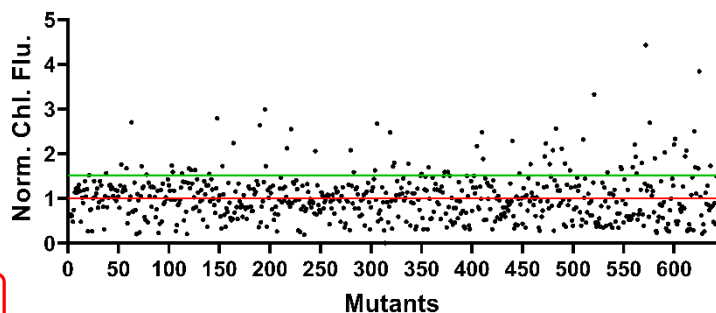**C**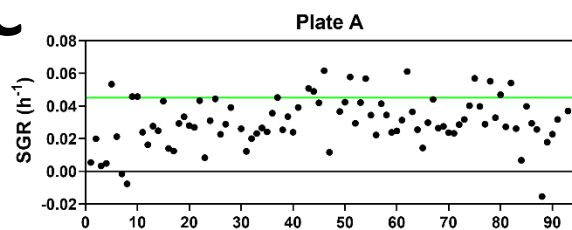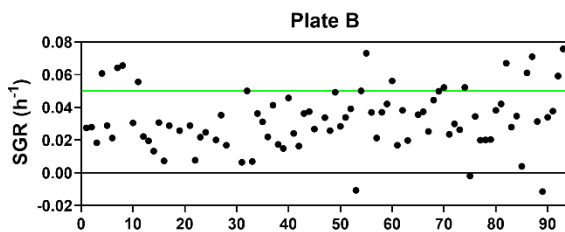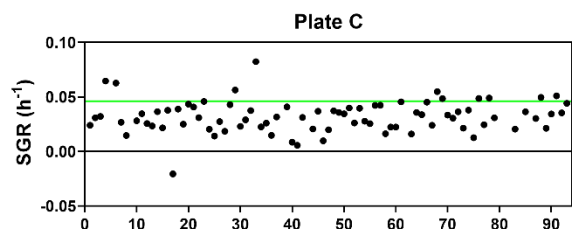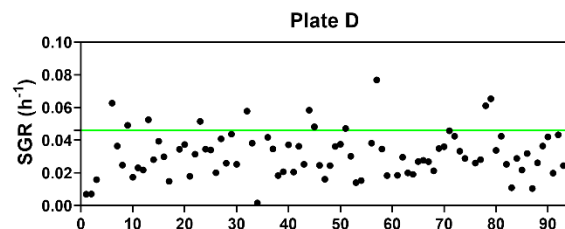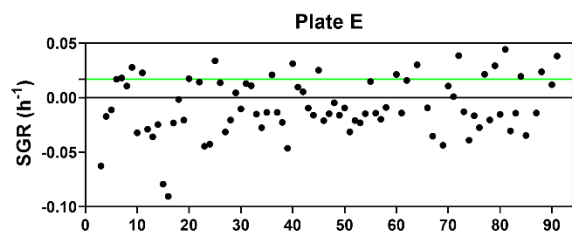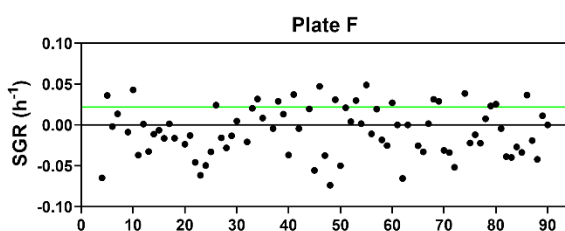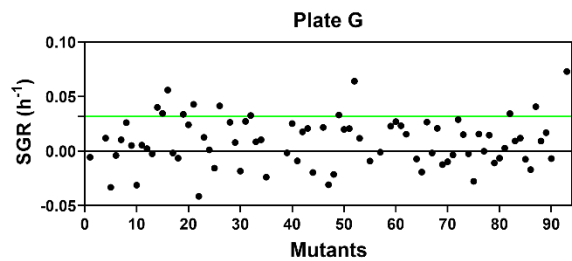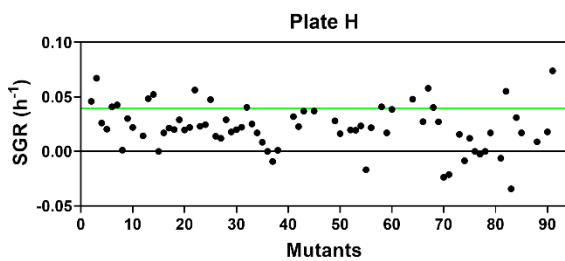**D**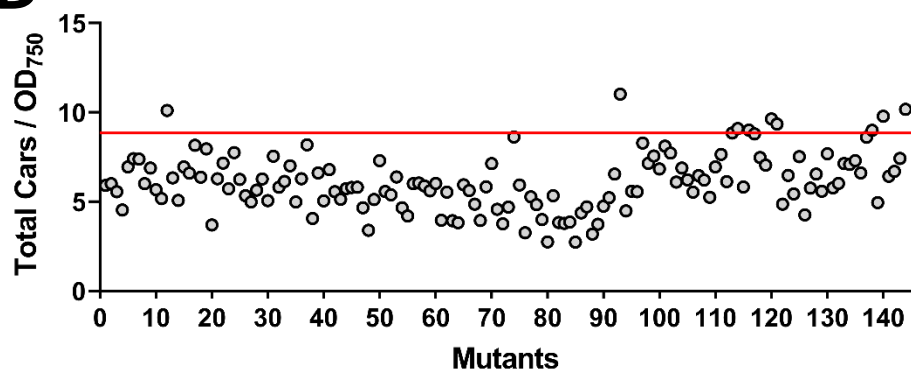

Supplement: Supplementary file 1 — Additional file 1: Lutein hyperaccumulating mutant screening and selection workflow. A Decision tree for initial round of mutant screening. SGR, specific growth rate; mean, average value for microplate on which mutant was grown; SD, standard deviation; ChlFlu, chlorophyll fluorescence; Y, yes; N, no. Strains that fit the criteria for the green boxes were sub-cultured into 24-well plates for the second round of screening. B Normalized chlorophyll fluorescence of mutant strains grown for first round of selection. Chlorophyll fluorescence measurements taken by plate reader after 4 days’ growth with the following parameters: excitation 440 nm, emission 680 nm, gains 50. Chlorophyll fluorescence readings for each mutant were normalized to 1/average fluorescence reading for its respective microplate. Each black dot represents an individual mutant strain in an individual well of a 96-well plate. Red line shows the average chlorophyll fluorescence normalized to 1; green line shows the average chlorophyll fluorescence for each plate + 1 standard deviation. B Specific growth rates of mutants grown for first round of selection. Specific growth rates were calculated from chlorophyll fluorescence measurements taken by plate reader for each well between Days 1 and 2. Each black dot represents an individual mutant strain in an individual well of a 96-well plate. Green line represents the average mutant growth rate per plate + 1 standard deviation. D Total carotenoid (Cars) content of 144 mutant C. reinhardtii strains adjusted to OD750. Total carotenoids were calculated following pigment extraction in pure acetone and subsequent spectrophotometer analysis [40]. Total carotenoid contents were adjusted to cell density at OD750. Each circle represents an individual mutant strain. Red line shows average total carotenoid value/ OD750 for control strain CC-125. [file 13068_2023_2421_MOESM1_ESM.pdf]

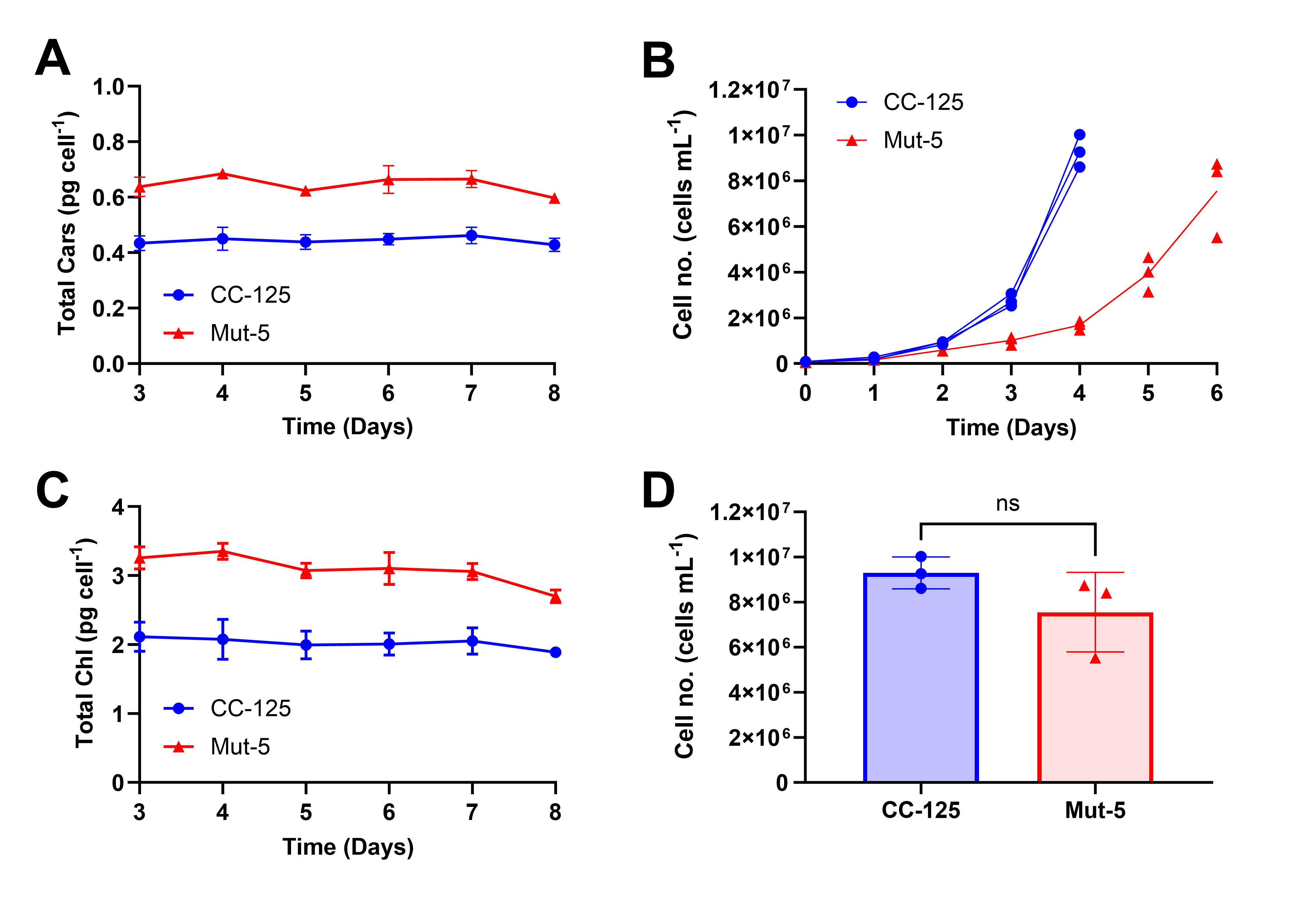

Supplement: Supplementary file 2 — Additional file 2: Pigment and growth data for proteomics time-point selection and cultures. A Total carotenoid (Cars) and C total chlorophyll (Chl) contents were measured daily for CC-125 and Mut-5 grown under standard conditions on Days 3–8, and are expressed here in pg per cell. Pigment concentrations were estimated using previously described extinction coefficients following acetone extraction and spectrophotometer analysis [40]. B Growth curves of samples harvested for proteomics analysis. D Final cell density measurements (in cells per mL) for the CC-125 and Mut-5 cultures harvested for proteomics analysis, between which there was no significant difference (p = 0.1888; Student’s t-test). [file 13068_2023_2421_MOESM2_ESM.tif]

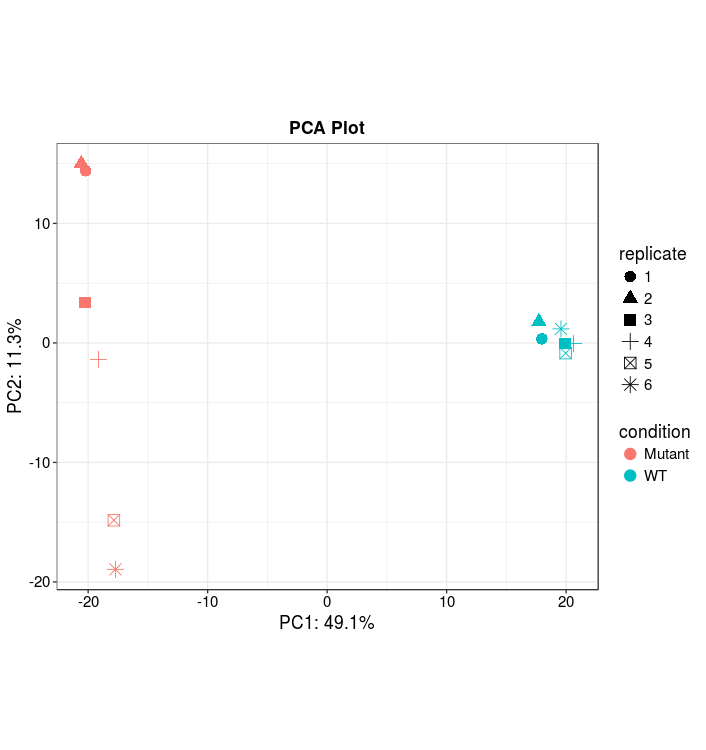

Supplement: Supplementary file 3 — Additional file 3: Principle component analysis of the Mut-5 vs CC-125 comparative proteomics data. Principle component analysis (PCA) plot showing six technical replicates of both CC-125 (blue) and Mut-5 (orange). The two strains cluster separately along the PC1 axis, indicative of proteomic differences between the two strains. [file 13068_2023_2421_MOESM3_ESM.png]
